# Recent Advances in ROS-Sensitive Nano-Formulations for Atherosclerosis Applications

**DOI:** 10.3390/pharmaceutics13091452

**Published:** 2021-09-11

**Authors:** Hao Ji, Renyi Peng, Libo Jin, Jiahui Ma, Qinsi Yang, Da Sun, Wei Wu

**Affiliations:** 1 Institute of Life Sciences & Engineering Laboratory of Zhejiang Province for Pharmaceutical Development of Growth Factors, Wenzhou University, Wenzhou 325035, China; 20180031@wzu.edu.cn (H.J.); 20170032@wzu.edu.cn (R.P.); 20160121@wzu.edu.cn (L.J.); 204513344001@wzu.edu.cn (J.M.); 2 Wenzhou Institute, University of Chinese Academy of Sciences, Wenzhou 325000, China; yangqs@wiucas.cn; 3 Key Laboratory for Biorheological Science and Technology of Ministry of Education, State and Local Joint Engineering Laboratory for Vascular Implants, Bioengineering College of Chongqing University, Chongqing 400030, China

**Keywords:** reactive oxygen species, nano-formulations, atherosclerosis, imaging, polymer

## Abstract

Over the past decade, ROS-sensitive formulations have been widely used in atherosclerosis applications such as ROS scavenging, drug delivery, gene delivery, and imaging. The intensified interest in ROS-sensitive formulations is attributed to their unique self-adaptive properties, involving the main molecular mechanisms of solubility switch and degradation under the pathological ROS differences in atherosclerosis. This review outlines the advances in the use of ROS-sensitive formulations in atherosclerosis applications during the past decade, especially highlighting the general design requirements in relation to biomedical functional performance.

## 1. Introduction

Cardiovascular diseases (CVDs) are the leading cause of death and major medical problems in the world, being responsible for more than 40% of the deaths globally from noncommunicable diseases [1,2]. Atherosclerosis is responsible for the main pathological basis of the high morbidity and mortality in patients with CVDs [3]. Atherosclerosis is a localized arterial disease caused by plaque buildup in the intimal layer of arteries, and it often occurs in the aorta, carotid, coronary, and peripheral arteries, which is characterized by lipid deposition, macrophage foaming, formation of atherosclerotic plaques, thickening and hardening of the vessel wall, and triggering of subsequent cardiovascular events [4]. Although the symptoms are usually not apparent at the early stage, atherosclerosis gives rise to various severe acute events and complications including coronary heart disease, hypertension, chronic kidney, stroke, and intermittent claudication at the advanced stage [5,6,7,8]. Furthermore, atherosclerosis is considered to be an important feature in CVD epidemiology and includes two major conditions: ischemic heart disease and stroke, which were the world’s second and third leading causes of death in 2019, respectively, causing 16% and 11% of all-cause mortality. Since 2000, the number of atherosclerosis CVDs has risen by more than 2.7 million to 9.6 million deaths with a growing burden for healthcare systems [9,10].

**Figure 1:**
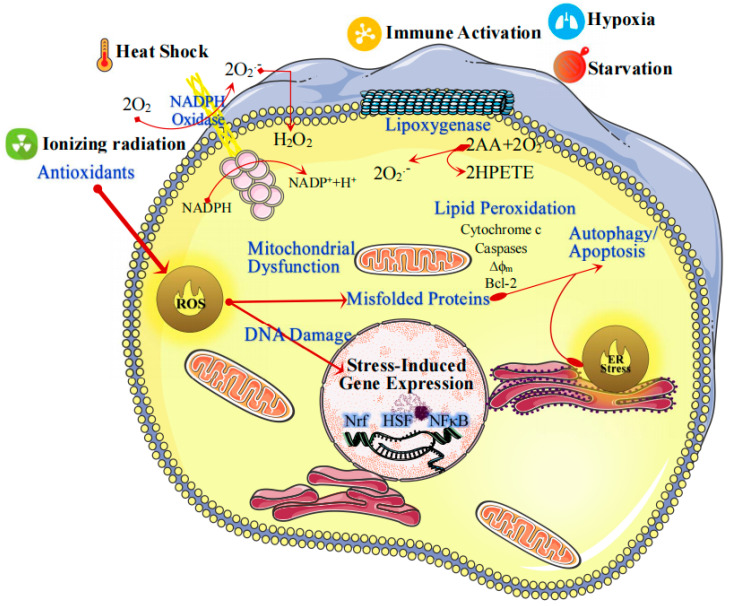
The determinants and consequences of oxidative stress. Oxidative stress causes an imbalance in free-radical formation through multiple pathways which can trigger programmed cell death pathways, and it has been linked to clinically relevant diseases [22,23,24].

In particular, reactive oxygen species (ROS), oxygen-derived metabolites including hydrogen peroxide (H_2_O_2_), superoxide, and hydroxyl radicals (•OH) [11,12], play a central role in atherosclerosis from its initiation and progression to acute thrombotic complications [13]. When there is a high level of blood lipids, the passage of lipids through the walls of blood vessels cannot be effectively blocked by endothelial cells, which stimulates ROS production in neutrophils [14]. Subsequently, the accumulation of ROS promotes the oxidation of low-density lipoprotein (LDL) to form the oxidized low-density lipoprotein (Ox-LDL), which is phagocytized by macrophages to produce deteriorated foam cells [15]. Moreover, the high content of ROS in atherosclerosis endothelial injury can stimulate macrophages to produce proinflammatory cytokines and chemokines, as well as intensify the inflammatory activity of macrophages [16,17]. Excessive ROS accumulation also induces vascular cell damage, inflammatory cell recruitment, lipid peroxidation, metalloproteinase activation, and extracellular matrix deposition, which collectively lead to vascular remodeling [18,19]. Therefore, ROS have vital effects on the mediation of several physiological processes, such as cell differentiation and proliferation, cellular metabolism, survival, and immune response (Figure 1) [20,21].

Despite great advances in diagnostic and therapeutic technologies for AS, the overall grim statistics of atherosclerosis have not significantly improved over the last decades, and they are estimated to continuingly increase [25,26]. In clinical practice, systematical blood lipid reduction and/or local anti-inflammation are the common strategies for atherosclerosis management. However, several challenges, such as low bioavailability, slow therapeutic effect, and serious side effects should be paid great attention [6,27]. To address these drawbacks, the ideal approach for effective and safe atherosclerosis management can involve target drug delivery, which has recently been a hot research topic in biomedical nano-formulations. Nano-formulations can facilitate cargo metabolism to improve the bioavailability and ease the liver burden from the high dose of drugs [28]. In particular, stimulus-sensitive nano-formulations are capable of accelerating cargo release under specific trigger conditions, such as ROS, pH, temperature, and enzymes [29,30,31]. Responding to the pathological ROS differences, desirable multifunctional carriers are able to reveal great changes in solubility and/or cleavable bonds in the molecular structures for “on-demand” cargo release at the atherosclerosis lesion [13,32,33,34,35,36,37,38]. Thus, pharmacological targeting of ROS-sensitive nano-formulations represents a promising therapeutic avenue for safe and effective atherosclerosis management.

Based on the perspectives discussed above, recent studies have shown that such nano-formulations exhibit outstanding advantages in early diagnosis, treatment, and evaluation of therapeutic effects in atherosclerosis [39], mainly featuring the following aspects: (1) strengthening the stability during blood circulation, thus extending the half-life of cargo; (2) selectively delivering cargo to the pathological vascular lesion, even pathology-related cells; (3) enhancing the local cargo concentration at the atherosclerosis lesion to meet the demands of the effective concentration window for optimizing imaging sensitivity, resolution, accuracy, and/or therapeutic efficacy; (4) personalized and precise treatment by controlling cargo release at the favorable site and “on-demand” dosing trigged by the pathological stimuli, not only improving the bioavailability, but also reducing the undesirable toxicity for the normal cells, tissue, and organs, which is particular important for chronic atherosclerosis with long-term dosing. Herein, in this review, we systematically summarize the molecular mechanisms and focus on the advanced applications of ROS-sensitive nano-formulations for potential atherosclerosis management, including scavenging excessive ROS, controlling cargo release, and imaging. Furthermore, the future challenges in development and application are also explored.

## 2. ROS-Sensitive Functional Molecular Structures

**Figure 2:**
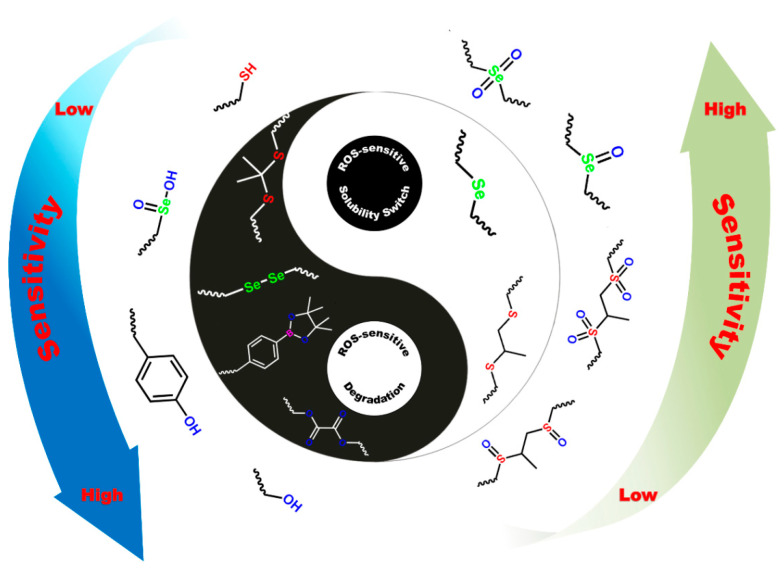
Two categories of ROS-responsive structure change and corresponding sensitivity of the polymeric biomaterials under oxidation stimulus.

Studies have shown that ROS are closely related to various pathological diseases, such as atherosclerosis, cancer, diabetes, and inflammation. Therefore, ROS accumulation can be used as specific stimuli to regulate the delivery and release of drug/gene vectors [34,40,41]. In general, ROS-sensitive functional molecular structures can be roughly divided into two categories: water solubility switch and structural degradation (Figure 2). Moreover, different ROS sensitivities are easy to construct depending on the various active bonds of structures, which is beneficial for providing flexible design strategies to meet demands in intricate pathological microenvironment applications.

### 2.1. Water Solubility Switch

#### 2.1.1. Polypropylene Sulfides (PPS)

Under oxidizing conditions, organic sulfur compounds change their hydrophilic or hydrophobic properties from hydrophobic sulfides to hydrophilic sulfones or sulfoxides [42,43]. In 2004, Hubbell first reported the use of polypropylene thioether as a drug delivery carrier, which was the first class of oxidization-sensitive responsive biomaterial [44]. They obtained ABA type triblock copolymers with polyethylene glycol as hydrophilic segment A and polypropylene sulfide as hydrophobic segment B via anionic ring-opening polymerization. PPS and polyethylene glycol (PEG) diblock copolymerization analogs [45] involved highly hydrophilic PEG self-assembled around the hydrophobic PPS core into nanoparticles with a particle size of about 20 nm, which could be effectively decomposed and release drugs in mouse model experiments. Because lymphocytes are able to actively utilize ROS as signaling molecules to regulate inflammation and oxidative stress, such ROS-responsive nanocarrier systems show great potential for the treatment of atherosclerosis [46].

#### 2.1.2. Selenium-Containing Block Copolymer

Similar to the thioether groups in PPS, selenium compounds also undergo a phase change in an oxidizing environment, from their initial hydrophobic state to hydrophilic soluble selenium oxide or selenium sulfone compounds [46]. Zhang synthesized a series of selenium-containing redox-responsive nano drug carrier materials [47,48,49,50]. Furthermore, Ma developed an amphiphilic selenium-containing triblock copolymer (PEG–USE–PEG) containing hydrophobic polyurethane and hydrophilic polyethylene glycol blocks [51].

#### 2.1.3. Polythioether Ketal

Among the multi-stimulus-responsivee materials developed today, polythioether ketal is one of the widely used ROS-sensitive structures [52,53]. Polythioether ketal polymer nanoparticles synthesized by Almutairi et al. were reported as having biological double stimulus-responsive properties and could be used as protein delivery carriers [52]. The thioether group in the material experienced a solution-change mechanism similar to PPS in response to ROS, while the ketal group had a pH response [53]. Nero red or ovalbumin were encapsulated in poly(thioether ketone) nanoparticles using a high-pressure homogenizer. When micelles enter macrophages with a high ROS concentration, the material is oxidized and dissolved, resulting in partial release of the coated material. In the presence of an acidic environment (pH = 6.5) and ROS in vitro, the polymer degrades almost completely to release the load [54].

### 2.2. Structural Degradation

#### 2.2.1. Boronic Esters

Borate ester compounds are biological materials with ROS-sensitive responsiveness and degradation, which have been widely used in recent decades. It was found that borate ester is oxidized and degraded in an ROS oxidation environment [40,55,56]. For example, covalently linking borates to peptides hides the active site of matrix metalloproteinase (MMP), exposing the active part to material degradation in a highly reactive oxygen environment. Similarly, when borates are covalently conjugated to anticancer drugs and imaging agents, the active components can also be activated in specific environments with high ROS levels to enhance tracer and therapeutic effects [57].

#### 2.2.2. Silicon

It was found that various drug molecules such as doxorubicin and dexamethasone adsorbed in porous silicon materials can be used for drug delivery therapy [58,59]. Under the stimulation of oxidation conditions, the matrix Si material is oxidized to form Si–O–Si bonds, before hydrolysis occurs to effectively release the payload. The experiment found that, in an oxidized environment simulated by peroxide nitrate (ONOO^−^), the fluorescence intensity expressed in the Si particles with covalently attached fluorescent dye within 24 h was 10 times that in normal saline, which effectively avoided the undesirable rapid release of the cargo [60].

#### 2.2.3. Proline Oligomers

Since 1960, free-radical-mediated oxidation of free amino acids or polypeptides has been extensively studied [53,61]. Its importance has been repeatedly emphasized recently because of the increased ROS levels in many pathological processes that oxidize proteins [53]. It was found that amino acids such as aspartic acid, glutamic acid, and proline in polypeptides were easily cleaved when oxidized, producing protein fragmentation. Among the three amino acids mentioned above, proline is known to be susceptible to oxidative cracking in the presence of physiological ROS [62]. Notably, unlike other species which quickly respond to ROS within hours to days, the complete degradation of oligomeric proline under ROS takes several weeks. Therefore, oligomeric proline -based carriers are the favorable choice for controlling the slow cargo release of oxygen in an inflammatory response for long-term dosing [63].

#### 2.2.4. Polythioketal

Thioketal is usually used as a protective group of carbonyl in organic synthesis and has good stability in acidic or alkaline environments [54]. It can be decomposed into ketones/aldehydes and disulfide compounds under oxidation conditions, which is a novel type of reactive oxygen chemical linkage. At first, Murthy’s group prepared thioketal nanoparticles via condensation polymerization for oral treatment of inflamed intestinal tissues with high reactive oxygen levels. Combined with siRNA, it can form load-bearing particles of about 600 nm, which can easily bind to inflammatory colon mucosa and be internalized into macrophages. These loaded particles are resistant to acids, bases, and proteases and are able to resist the harsh environments of the gastrointestinal tract for targeted delivery to inflammatory sites [64].

### 2.3. Other Types

In addition to the two categories of water solubility-switchable and structure-degradable structures, derivatives containing methionine [65], arylox-alate [66], vinyldithioether [67,68], and ferrocene [69] have also been explored for ROS-sensitive nano-formulation construction.

## 3. ROS-Sensitive Nano-Formulations for Atherosclerosis Applications

### 3.1. Nano-Formulations for Scavenging Excessive ROS

Introducing exogenous antioxidants into the biological system to scavenge excessive ROS is a feasible strategy to prevent oxidative stress and improve atherosclerosis. However, the clinical application of traditional antioxidants still faces numerous challenges, such as high toxicity, inefficient delivery, and short duration of drug residence. In recent years, antioxidant design strategies based on multifunctional nano-carriers have been proposed to construct ROS scavengers for the treatment of atherosclerosis. Using nano-carriers can improve the stability and bioavailability of traditional antioxidant drugs for targeted cargo delivery [31,70]. 

Ferulic acid is an attractive anti-atherosclerosis drug because it reduces macrophage adipogenesis and is effective in scavenging free radicals by forming a resonantly stable phenoxy radical [71]. In order to overcome the limitations of stability, dosing, and targeted delivery action of ferulic acid, Rebecca et al. designed a nanoparticle that chemically conjugates ferulic acid with poly(anhydride-ester) using an adipic acid or diglycolic acid linker [72]. This method can effectively protect ferulic acid from decarboxylation, enhance the total mass of ferulic acid in the polymer, and achieve a sustained and tunable release. After treatment with this nanoparticle, oxLDL uptake and ROS production by human monocyte-derived macrophages were significantly reduced, which is important for preventing the formation of macrophage foam cells [72]. D-α-Tocopherol, the active form of vitamin E, has multiple protective effects such as reducing oxidative lipids and scavenging ROS in cardiovascular diseases [73,74]. After combination with MnO_2_ microparticles, the nano-complex can inhibit progression of atherosclerosis by reducing the ROS levels and inhibiting the oxidation of LDL [75].

Recently, inorganic materials with intrinsic ROS-scavenging properties have been developed to decrease the damage induced by excessive ROS. These enzyme-like antioxidative nanoparticles defined as nanozymes exhibit high stability and biocompatibility; therefore, they have great potential for clinical application. MnO_2_ can catalyze the decomposition of hydrogen peroxide to produce water, oxygen, and Mn2^+^ ions, which has been explored for scavenging ROS in inflammatory conditions [76,77]. For example, Bizeau et al. prepared an MnO_2_ spherical microparticle coated with HA [75], which was biocompatible and had high scavenging capacities toward H_2_O_2_ in vitro studies [75]. CeO_2_ is an artificial superoxide dismutase with strong antioxidant properties as a potential free-radical scavenger [78]. Wu et al. synthesized two iron oxide (core)–cerium oxide (shell) nanoparticles (IO@CO1 and IO@CO2), which exhibited significant ROS-scavenging ability, enhanced macrophage uptake, and exhibited low cytotoxicity, showing great potential for the diagnosis and treatment of ROS-related inflammatory diseases. After treatment with 0.14 μg/mL of cerium oxide using IO@CO2 in J 774A.1 macrophages, 73% of ROS were scavenged, thereby significantly improving the pathological microenvironment [79]. Fullerenes and their derivatives also have intrinsic ROS-scavenging capabilities, which can react with free-radical species such as superoxide (O_2_-), •OH, and H_2_O_2_, for the treatment of neurodegenerative and inflammatory diseases [80,81]. However, their clinical application as antioxidant nanotherapies for atherosclerosis treatment is continuously improving, and the acute and long-term toxicities should be comprehensively investigated. 

**Figure 3:**
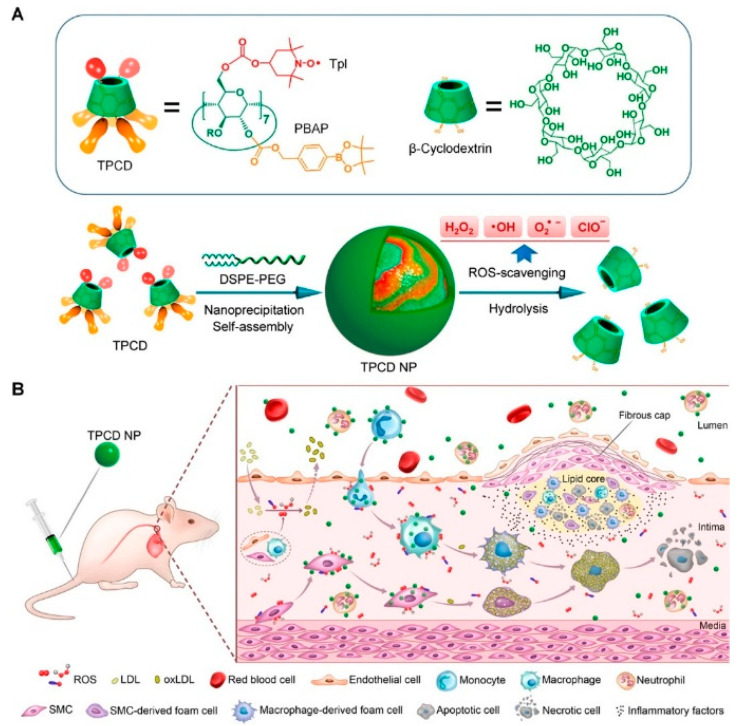
(**A**) Chemical structure and preparation of ROS-scavenging TPCD nanoparticles. (**B**) Schematic of targeted treatment of atherosclerosis in mouse model using TPCD nanoparticles. Adapted from [86].

Some polymers have also been exploited as antioxidants, and their ROS-quenching effect is mainly attributed to their special structures and inherent catalytic properties. An amphiphilic block copolymer, poly(ethylene glycol)–poly(tyrosine-ethyl oxalyl), was synthesized and self-assembled into micelles loaded with simvastatin, a kind of statin drug with antioxidant and anti-inflammatory effects [82,83,84]. This kind of micelle can consume ROS in pathologic sites and exert a synergistic antioxidant effect with simvastatin. Intravenous administration of micelles in ApoE^−/−^ mice can efficiently reduce the lesion area of plaque, showing great therapeutic effects on atherosclerosis [84]. Another case is represented by PBAPs that can be oxidized by H_2_O_2_ to generate phenolic compounds. The PBAP structure was employed as an H_2_O_2_-labile entity to synthesize H_2_O_2_-eliminating materials via covalent conjugation to biocompatible scaffold compound β-cyclodextrin. Importantly, the H_2_O_2_-scavenging capability was positively associated with the number of conjugated PBAP groups [85]. Hence, a broad-spectrum ROS-scavenging nanoparticle TPCD was further developed by simultaneously conjugating superoxide dismutase mimetic agent TEMPOL and PBAP to β-cyclodextrin (Figure 3) [86,87]. TPCD nanoparticles could effectively eliminate multiple reactive oxygen species after being rapidly endocytosed by macrophages and VSMCs. In vivo studies showed that TPCD nanoparticles effectively inhibited the atherosclerosis progression and stabilized plaque development by inhibiting ROS-induced inflammatory responses [86]. Furthermore, Guo et al. fabricated a novel nanoparticle with multiple pharmacological activities using luminol covalently conjugated to chemically modified β-cyclodextrin, which showed desirable therapeutic efficacy for the treatment of inflammatory diseases by inhibiting inflammatory response and oxidative stress, in addition to exhibiting a favorable safety performance in a mouse model [88]. 

### 3.2. ROS-Sensitive Nano-Formulations for Controlling Cargo Release 

Stimulus-responsive drug delivery systems can achieve local drug release by targeting spatiotemporal control sites, which is considered to be more reliable and effective for triggering drug release. In light of the high ROS levels in atherosclerosis lesions, ROS-sensitive materials can be designed to achieve atherosclerosis targeting and control anti-atherosclerosis drug release. Andro, a labdane diterpene, possesses remarkable anti-inflammatory and antioxidant properties by interfering with several transcription factors and signaling pathways such as AP-1, HIF-1, (NF)-κB, MAPK, and JAK/STAT [89,90]. Construction of a controlled drug carrier that releases andro in response to high levels of ROS has important implications for atherosclerosis treatment. Wu et al. designed an andro-loaded micelle that was assembled from the amphiphilic diblock copolymer PEG–PPS [14]. The hydrophobic block PPS can respond to ROS, subsequently switching into hydrophilic molecules. The changes in hydrophobic interaction can drive micellar decomposition, leading to drug release [91]. Interestingly, the ROS-sensitive switch in polymer hydrophobicity only occurs at pathological locations where the ROS concentration is much higher than that in normal tissues, thereby significantly improving the therapeutic efficacy. The micelle itself consumes ROS at the atherosclerotic lesion and acts as an ROS-responsive drug carrier for rapid andro release, thereby simultaneously alleviating inflammation and oxidative stress. After ApoE^−/−^ mice were treated with the andro-loaded micelle, almost no lipid deposits were found, showing its prominent therapeutic effect [14]. In another study, celastrol, a hydrophobic inhibitor targeting the NF-κB signaling pathway, was encapsulated into the same carrier, i.e., ROS-responsive PEG–PPS micelles; they were found to significantly enhance the therapeutic efficacy of NF-κB inhibition and decrease the undesirable cytotoxicity for normal cells and tissues both in vitro and in vivo [92]. Dou et al. constructed an ROS-responsive nanoparticle based on chemically modified β-cyclodextrin. Compared with the nonresponsive poly(lactide-*co*-glycolide) nanoparticle control group, the ROS-responsive nanoparticle could serve as a superior delivery vehicle for atherosclerosis therapy by selectively releasing rapamycin, responding to the abnormally high ROS stimulus at atherosclerotic lesions. Intravenous administration of this nanoparticle in ApoE^−/−^ mice could effectively attenuate plaque development and stabilize atheromatous lesions [93]. 

ROS-responsive functional groups are widely involved in nanomedicines, which have been successfully used in the research of diagnosis and treatment of atherosclerosis, ischemia reperfusion injury, and tumors [94,95]. The functional groups of thioketal linkages and ferrocene were used to design a dual ROS-sensitive nanoparticle and CD44 receptors targeting carrier material HASF. Curcumin, a traditional anti-inflammatory and antioxidant drug, was encapsulated by self-assembly to obtain HASF@Cur micelles [96,97]. Drug release profiles in vitro indicated that curcumin was effectively released from micelles trigged by the high levels of ROS, and the release rate was positively correlated with H_2_O_2_ concentration. More importantly, treatment with HASF@Cur micelles in vivo significantly inhibited the average lesion area, showing a favorable therapeutic effect compared to free curcumin [96].

Recently, cell-derived biomimetic nanoparticles have attracted extensive attention due to their long-term blood circulation, lower immunogenicity, and enhanced target capability compared to traditional nanoparticles [98]. In recent studies, biomimetic delivery systems derived from macrophage membranes were developed to encapsulate ROS-responsive nanoparticles for atherosclerosis treatment [38,99,100]. These cellular function-driven approaches can achieve targeted delivery to inflammatory tissues without resorting to additional target molecules, as well as reduce the clearance of nanoparticles by the reticuloendothelial system. After accumulation at the targeted inflammatory sites, anti-atherosclerotic drugs loaded in ROS-sensitive nanoparticles would be subsequently released responding to the high concentration level of ROS, thereby enabling effective drug therapy [99]. Moreover, in our previous study, a biomimetic and intelligent nanosystem was designed using ROS-responsive 5-aminolevulinic acid encapsulated with rapamycin, which were coated with a red blood cell membrane [101]. This biomimetic nano-formulation provided a feasible method to prevent the premature release of drugs during circulation, as well as break the shell in response to high H_2_O_2_ exposure, thus having potential therapeutic effects toward atherosclerosis. In a recent study, considering the critical role of macrophages during the development of atherosclerosis, we also employed macrophage membranes to camouflage the ROS-responsive nanoparticles loaded with rapamycin for atherosclerosis treatment. This biomimetic strategy could facilitate nanoparticle escape from macrophage clearance, thereby prolonging blood circulation time. Moreover, this biomimetic nano-formulation had a favorable biocompatibility and significantly inhibited the proliferation of macrophages in an in vitro study, showing potential application prospect for atherosclerosis treatment [38]. 

Shen et al. developed a system that responds to both ROS and shear stress in atheromatous plaques, consisting of red blood cells and PGED–PPS micelles [102]. These micelles can be adsorbed onto the surface of red blood cells via electrostatic incorporation, resulting in the extended circulation longevity of nanoparticles. Hydrophobic PPS is readily disassembled in response of excess ROS, thereby accelerating the loaded simvastatin release for the synergistic anti-atherosclerosis efficiency of drugs and bioactive carriers. In vivo studies showed that the application of this delivery system is capable of sustaining long-term release of simvastatin, effectively prolonging its half-life.

In addition to various kinds of ROS-responsive nano-formulations for delivering traditional anti-atherosclerosis drugs, gene delivery for atherosclerosis therapies based on ROS-responsive nano-carriers has received intensive attention. Gupta et al. developed a safe and effective vascular-targeted gene delivery tool to improve plasmid DNA transfection by synthesizing a dual ROS- and pH-responsive nanocarrier [62]. This oligo-proline peptide-derived nanocarrier with ROS-sensitive degradability performs a specific functional application only under high ROS conditions. In an in vitro study, the application of this gene delivery tool promoted the cellular uptake and expression of the luciferase reporter gene in an ROS-rich environment [62].

**Figure 4:**
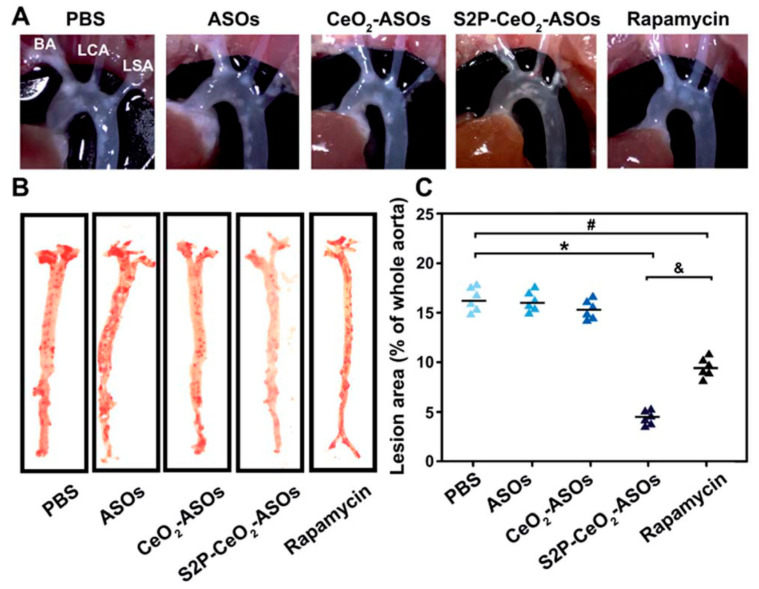
(**A**)The atherosclerosis efficacy of indicated materials following intravenous administration into ApoE^−/−^ mice Representative images of the in situ aortic arch lesions at the brachiocephalic artery (BA), left carotid artery (LCA), and left subclavian artery (LSA). (**B**) Representative images of oil red O-stained *en face* aorta. (**C**) The lesion area as a percentage of the whole aorta. * *p* < 0.05 for S2P-CeO2-ASOs vs. PBS, # *p* < 0.05 for rapamycin vs. PBS, & *p* < 0.05 for S2P-CeO2-ASOs vs. rapamycin. Adapted from [105].

RNAi using ASOs or siRNA has been developed, showing promising potential for the treatment of intractable diseases including atherosclerosis [103]. Packaging ASOs or siRNAs into nanoparticles can avoid the degradation of nucleases, prolong the half-life in circulation, and improve targeted delivery and release. mTOR plays an important role in the initiation and progression of atherosclerosis by controlling autophagy and lipid metabolism, representing a promising target site for atherosclerosis treatment [104]. Gao et al. designed an H_2_O_2_-sensitive ASO delivery nanoplatform (S2P-CeO2-ASOs) to efficiently silence mTOR and rescue the impaired autophagy [105]. They used S2P to enhance plaque targeting and penetration, the PEG segment to prolong the blood circulation time in vivo, and the CeO_2_ core to facilitate carrier escape and ASO release in an “on-demand” manner, responding to H_2_O_2_. Following intravenous administration into ApoE^−/−^ mice, the S2P-CeO2-ASO nano-formulation induced more than 75% of the mTOR expression knockdown in aortas, thus showing its superior therapeutic efficacy for inhibiting the progression of atherogenesis (Figure 4) [105]. Furthermore, Hou et al. developed a hyaluronan-based ROS-responsive nano-formulation that could co-deliver the anti-inflammatory drug dexamethasone and siRNAs for targeting mTOR therapy. In vitro transfection studies of this co-delivery strategy on RAW264.7 cells exhibited an excellent gene silencing effect on the mTOR gene and decreased the mRNA expression of proinflammatory cytokines, showing a synergistic anti-inflammatory effect [106].

### 3.3. ROS-Sensitive Nano-Formulations for Imaging 

**Figure 5:**
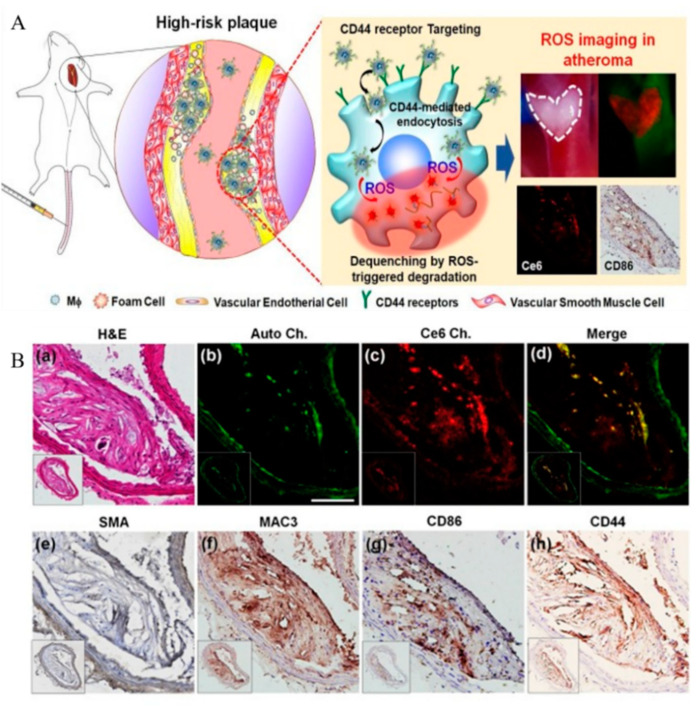
(**A**) Schematic of ROS imaging of proinflammatory macrophages within atherosclerotic plaques. (**B**) Histological and immunohistochemical evaluations. (**a**) H&E staining. (**b**) Autofluorescence signals from elastin (green), (**c**) NIRF signals (red), and (**d**) merged image. Immunohistochemical staining of (**e**) SMA, (**f**) MAC3, (**g**) CD86, and (**h**) CD44. Scale bar: 25 mm. Adapted from [109].

In addition to the targeted treatment of atherosclerotic plaques, the in vivo traceability of ROS-responsive nano-agents has been used for the development of novel protocols for atherosclerotic diagnosis and monitoring. Recently, Wang et al. developed an early detection method for targeting foam cells in atherosclerosis. A platelet membrane was engineered to encapsulate two naphthalimide-based fluorescent probes to detect ROS in foam cells. One of their obtained nano-detection systems, TBNG@Mp, showed low toxicity and exhibited fluorescence imaging in the thoracic aorta of early atherosclerosis model rats [107]. To further quantify ROS production in the vasculature and various tissues in atherosclerosis, Manea et al. developed an imaging nano-formulation that encapsulated a redox-sensitive fluorescent probe, ROS Brite^TM^700, using PEG-stabilized liposomes (Lp), which could selectively recognize the highly expressed VCAM-1 to exhibit NIRF upon ROS oxidation, prior to further analysis using a high-resolution imaging system for ROS quantification [108]. Recently, Park et al. developed an NIRF nano-sensor using HA as a ligand for the CD44 receptor, chlorin e6 as an NIRF dye, and a thioketal linker as an ROS-degradable moiety [109]. The in vivo study indicated that the nano-sensors were internalized into proinflammatory macrophages and were then cleaved selectively by the intracellular ROS stimulus, resulting in the pronounced recovery of fluorescence signals for imaging the ROS in atherosclerotic plaques (Figure 5) [109].

ROS-sensitive photodynamic therapy agents can be designed as innovative theranostic platforms for target imaging and atherosclerosis therapy. Hyaluronic acid, an anionic polysaccharide, is one of the representative biomaterials used as a cleavable substrate for selective ROS detection [110,111]. Kim et al. synthesized MacTNPs by conjugating photosensitizer chlorin e6 to HA [112]. The fluorescence of MacTNPs is inhibited in the native state because of the self-quenching effect of conjugated chlorin e6. However, when MacTNPs are internalized into macrophages, the excess ROS cleaves the chemical bonds of HA, causing chlorin e6 release and resulting in fluorescence emission [112]. This ROS-activatable photosensitizing agent has a high target-to-background ratio and minimal side effects, implying its great potential in NIRF imaging and photodynamic atherosclerosis therapy.

**Figure 6:**
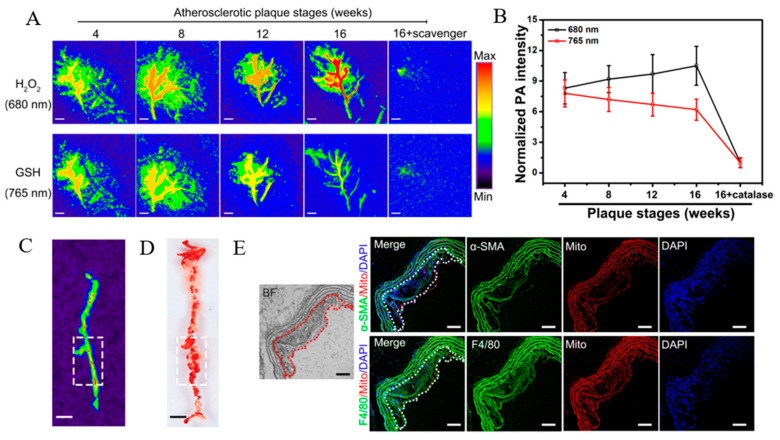
(**A**) PA imaging of atherosclerotic plaques in ApoE^−/−^ mice injected with BSA-Cy-Mito at the indicated stages. (**B**) Normalized PA intensities of plaques at different stages. (**C**) PA imaging at plaque lesions across the aorta of ApoE^−/−^ mice. (**D**) *En face* aortic lesions in Oil red O-stained aorta. (**E**) Fluorescence microscope images of aortic plaques in ApoE^−/−^ mice. Scale bar = 100 μm. Adapted from [114].

Photoacoustic imaging is the latest imaging technique, which integrates the advantages of ultrasound and optical imaging. Its principle is to detect the ultrasonic waves generated by the energy transduction of laser through thermoelastic expansion [113]. Gao et al. constructed a photoacoustic imaging nanoprobe using a GSH/H_2_O_2_ redox couple as the primary target for the imaging of redox status changes to assess the vulnerability of atherosclerotic plaques [114]. Two types of NIRF probes, the Cy-3-NO_2_ response to GSH and the Mito-NIRHP response to H_2_O_2_, were introduced to functionalize BSA, harvesting a BSA-Cy-Mito nanoprobe. In vivo results demonstrated that the BSA-Cy-Mito nanoprobe showed excellent photoacoustic imaging efficacy with high specificity and sensitivity, yielding insight into the GSH/H_2_O_2_ redox-related inflammatory process (Figure 6) [114]. 

Fluorescent groups with TP excitation and the AIE effect would be suitable materials as nanoparticle tracers, as well as for the spatial fluorescence imaging of inflammatory tissues, because of the clearer size resolution, less auto-fluorescence interference, and stronger imaging penetration [115,116,117]. Recently, Ma et al. combined two-photon AIE bioimaging and ROS-responsive drug delivery for the diagnosis and therapy of inflammation [118]. Prednisolone, a widely used anti-inflammatory glucocorticoid [119], was linked to the TP using an ROS-responsive bond to form a diagnosis therapy compound TPP, which was then encapsulated into the amphipathic polymeric PMPC–PMEMA micelles to obtain TPP@PMM. Once TPP@PMM was accumulated at inflammatory tissues, the aggregated micelles exhibited significant two-photon fluorescence emission and subsequently triggered micelle disintegration for precise drug delivery under the concentrated ROS stimulus [118]. Moreover, an improved nano-platform with simultaneous response to the high concentration of ROS and enriched lipids at the atherosclerosis site was developed. By conjugating TP to β-cyclodextrin carrying prednisolone, the compound TPCDP was synthesized, and it was then packaged into PMM to obtained TPCDP@PMM [120]. The in vivo bioimaging of TPCDP@PMM on ApoE^−/−^ mice showed efficient accumulation in atherosclerosis tissue and clear plaque recognition. In addition, the development of atherosclerotic plaques was effectively inhibited by the “two-pronged” anti-inflammatory and lipid removal approach, revealing their great potential for atherosclerosis theranostic applications [120]. 

## 4. Conclusions and Future Perspectives

ROS-responsive drug delivery systems have tremendous advantages in the diagnosis and treatment of many diseases, and they are even more significant in oxidative stress-related diseases. Due to the abnormal level of ROS in pathological atherosclerosis lesions, ROS-sensitive nano-formulations can be widely applied in target drug delivery and controlled cargo release for promoting bioavailability and reducing side effects, along with broad application prospects. Hydrophobic–hydrophilic phase transitions and cleavable bonds under pathological ROS stimulus are widely used to prevent premature cargo release and undesirable delivery for smart polymeric nano-formulation construction. For atherosclerosis applications, ROS-sensitive nano-formulations have been designed for scavenging excessive ROS in lesions, controlling cargo release in response to the pathological ROS stimulus, and imaging for atherosclerosis diagnosis, which provides a great platform for improving atherosclerosis applications. On the other hand, there is a long way before ROS-sensitive formulation development leads to clinical applications in atherosclerosis. ROS typically changes dynamically in terms of species and concentration as a function of location, stage, and disease. The balance between ROS sensitivity and stability is paramount for enhancing the ultimate efficacy of therapy and imaging and reducing the undesirable side effects. Further development addressing the key challenges will greatly enhance potential applications in atherosclerosis.

## Data Availability

Not applicable.

## Abbreviations

OH	hydroxyl radicals
andro	andrographolide
AIE	aggregation-induced emission
ASOs	antisense oligonucleotides
BSA	bovine serum albumin
CeO_2_	cerium oxide
CVDs	cardiovascular diseases
H_2_O_2_	hydrogen peroxide
HA	hyaluronic acid
HASF	oligomeric hyaluronic acid-2′-[propane-2,2-diyllbls(thio)] diacetic acl-hydroxymethylferrocene
LDL	low-density lipoprotein
Lp	liposomes
MacTNP	macrophage-targeted theranostic nanoparticles
MMP	matrix metalloproteinase
MnO_2_	manganese dioxide
mTOR	mammalian target of rapamycin
NIRF	near-infrared fluorescence
O_2_^-^	superoxide
ONOO^−^	peroxide nitrate
Ox-LDL	low-density lipoprotein
PBAPs	phenylboronic acid pinacol esters
PEG	polyethylene glycol
PEG–PPS	poly(ethylene glycol)–poly(propylene sulfide)
PGED–PPS	poly(glycidyl methacrylate)–polypropylene sulfide
PPS	polypropylene sulfides
RNAi	RNA interference
ROS	reactive oxygen species
S2P	stabilin-2-specific peptide ligand
siRNA	short interfering RNA
TEMPOL	4-hydroxy-2,2,6,6-tetramethylpiperidine-1-oxyl
TP	two-photon fluorophore
TPCD	superoxide dismutase mimetic agent TEMPO and PBAP in β-cyclodextrin
VSMCs	vascular smooth muscle cells
